# Insight of Melatonin: The Potential of Melatonin to Treat Bacteria-Induced Mastitis

**DOI:** 10.3390/antiox11061107

**Published:** 2022-06-02

**Authors:** Hongyang Li, Peng Sun

**Affiliations:** State Key Laboratory of Animal Nutrition, Institute of Animal Science, Chinese Academy of Agricultural Sciences, Beijing 100193, China; 15840076108@163.com

**Keywords:** mastitis, melatonin, immune response, antioxidant, anti-inflammatory

## Abstract

Bovine mastitis is a common inflammatory disease, mainly induced by bacterial pathogens, such as *Staphylococcus aureus*, *Escherichia coli,* and *Streptococcus agalactiae*. Mastitis has negative effects on the production and quality of milk, resulting in huge economic losses. Melatonin, which is synthesized and secreted by the pineal gland and other organs, is ubiquitous throughout nature and has different effects on different tissues. Melatonin is crucial in modulating oxidative stress, immune responses, and cell autophagy and apoptosis, via receptor-mediated or receptor-independent signaling pathways. The potent antioxidative and anti-inflammatory activities of melatonin and its metabolites suggest that melatonin can be used to treat various infections. This article reviews the potential for melatonin to alleviate bovine mastitis through its pleiotropic effect on reducing oxidative stress, inhibiting pro-inflammatory cytokines, and regulating the activation of NF-κB, STATs, and their cascade reactions. Therefore, it is promising that melatonin supplementation may be an alternative to antibiotics for the treatment of bovine mastitis.

## 1. Introduction

Bovine mastitis is a common disease, affecting the dairy industry worldwide [1,2,3]. Mastitis results in enormous economic losses, not only due to the increased costs of treatment, preventive measures, and additional workers, but also due to reduced profits resulting from the lower quantity and quality of milk. The increased number of infected cattle has resulted in mastitis becoming the main impediment to the development of the dairy industry [3].

Two types of mastitis have been described: clinical mastitis and subclinical mastitis [4,5]. Clinical mastitis results in visible changes in mammary glands and milk, including the swelling and/or bleeding of mammary tissue and the occurrence of clots and/or flakes in milk [5,6]. Subclinical mastitis is regarded as more harmful because it cannot be diagnosed by apparent symptoms. The diagnosis of subclinical mastitis requires further tests, such as determining somatic cell count (SCC) or the yield of milk [5,7]. Both types of mastitis have a severe and direct negative impact on bovine health and the dairy industry [8].

Antibiotics are shown to be effective in the treatment of mastitis [9,10]. However, invading pathogens may develop a resistance to antibiotics, and residual antibiotics may be present in milk consumed by humans. These possible deleterious side effects on bovine and human health reduced the use of antibiotics to treat bovine mastitis [9,10], emphasizing the importance of developing efficient therapeutic agents for use in dairy farming.

Melatonin, also known as N-acetyl-5-methoxytryptamine, is mainly synthesized in and secreted by the pineal gland. In mammals, melatonin is synthesized from L-tryptophan via four major enzymatic steps: hydroxylation, decarboxylation, acetylation, and methylation [11]. In the first step, tryptophan hydroxylase catalyzes the hydroxylation of L-tryptophan on its indole ring, yielding 5-hydroxytryptophan. Subsequently, 5-hydroxytryptophan is decarboxylated by pyridoxal phosphate and 5-hydroxytryptophan decarboxylase to produce serotonin, which is converted to *N*-acetylserotonin by acetyl-coenzyme A and arylalkylamine N-acetyltransferase (AANAT), the rate-limiting enzyme in melatonin synthesis. Finally, the hydroxl group of *N*-acetylserotonin is methylated by hydroxyindole-*O*-methyltransferase and *S*-adenosyl methionine to yield melatonin. The plasma concentration of plasma melatonin varies over a 24 h period, being higher during the night and lower during the daytime [12]. This cyclic production is regulated by the suprachiasmatic nucleus [13]. Melatonin secretion is regulated by a polysynaptic pathway, which modulates the circadian release of norepinephrine from sympathetic nerve fibers during the night [14]. Norepinephrine activates β1-adrenergic receptors on pinealocytes, initiating a specific molecular sequence that enhances intracellular cAMP concentration. The increase and subsequent activation of cAMP-dependent protein kinase A (PKA) are indispensable for the stimulation of AANAT [12]. The phosphorylation of AANAT protects it from protease hydrolysis, thereby promoting the synthesis of melatonin. The lack of norepinephrine-mediated stimulation during the day results in the suppression of melatonin levels by the rapid proteolytic decomposition of AANAT. Indeed, melatonin is synthesized by tissues other than the pineal gland, such as the retina [15], skin [16], gastrointestinal tract [17], thymus [18], lymphocytes [19,20], and bone marrow [21], with this synthesis modulated by paracrine or autocrine mechanisms rather than the circadian cycle [22]. Based on its ubiquitous distribution in animal cells and organs, it is not surprising that melatonin triggers various functions through many molecular pathways. Melatonin plays crucial roles in modulating physiological and biological activities, including sleep patterns [23,24] and circadian rhythms [25,26], reproduction [27,28] and sexual behavior [29,30], immune response [31,32] and enhancement [33,34], cell apoptosis [35] and proliferation [36], tumor occurrence and development [37,38], aging [39] and antioxidant processes [40], glucose [41,42] and lipid metabolism [43,44], inflammation [45], and angiogenesis [46,47].

Melatonin also shows pleiotropic effects, depending on both receptor-mediated and receptor-independent pathways [48,49]. Melatonin receptors include members of the nuclear receptor RORα/RZR family and the membrane receptors MT1 and MT2, which belong to the G protein-coupled receptor superfamily [50,51,52]. Generally, melatonin function is activated by its binding to high affinity receptors, triggering multiple signal pathways via a cascade effect [53,54]. In addition to having membrane and nuclear receptors, melatonin is reported to bind to various cytoplasmic [55] and mitochondrial [56] binding proteins, including quinone reductase-2 [57], calmodulin [58], and calreticulin [59]. Despite many studies on melatonin activity, few have assessed whether melatonin is effective for treating mastitis. This review evaluates whether melatonin can alleviate and treat bacteria-induced mastitis, enhancing bovine mammary health.

## 2. The Pathogenesis of Bacteria-Induced Mastitis

Factors associated with the development of mastitis include the physiological status of the animal [60], environmental hygiene [61], bacteria [62], and viruses [63], with bacteria regarded as the most frequent cause of mammary gland inflammation. The three major types of bacterial pathogens causing mastitis are *Staphylococcus aureus*, *Escherichia coli* and *Streptococcus*. *S. aureus* is the principal cause of subclinical mastitis in humans [64] and animals, including dairy cattle [65,66,67,68]. Mastitis induced by *S. aureus* has a negative worldwide impact on animal welfare [69,70], food safety [71,72], and productivity [73,74]. Although clinical and subclinical inflammation show long-term persistence, the validity of vaccination against *S. aureus*-induced mastitis has not been proven. Therefore, cattle are susceptible to recurrent infections, with incurable individuals are eventually eliminated. The two major causes of clinical mastitis in dairy cattle, *E. coli* and *Streptococcus*, significantly increase somatic cell count [75,76,77] and significantly decrease milk yield [78,79,80]. Valid methods are therefore needed to reduce the incidence of inflammation and the duration of infection.

Although the pathogenesis of mastitis has not been completely determined, oxidative stress [81,82,83] and autophagy [84] may be involved in this type of inflammation. This type of inflammatory process is generally coupled with oxidative stress reactions [85]. For example, *S. aureus* invasion and replication within cells would produce a large number of reactive oxygen species (ROS), subsequently disrupting redox homeostasis [86]. Although appropriately increased oxidative stress is necessary to kill pathogenic microorganisms, excess free radicals would stimulate host cells, resulting in the release of pro-inflammatory cytokines, such as tumor necrosis factor alpha (TNF-α) [87]. TNF-α is reported to destroy various intracellular molecules, inducing lipid peroxidation (LPO); these ROS subsequently injure mammary tissues by damaging cell membranes [87]. They may also damage the structure of DNA and proteins, resulting in dysfunctional cells and tissues, ultimately threatening animal health [87].

Autophagy, a highly strict systematic process, is considered a protector in resisting various stimuli and environmental damage and plays an important role in regulating normal cell physiology [88]. In other words, autophagy can be activated in response to the pernicious stress of the cell. It is reported that autophagy possesses a crucial degradation mechanism within cells that participates in the elimination of invading pathogenic microorganisms via interactions between these pathogenic microorganisms and autophagy receptors [88,89]. Because impaired autophagy is involved in bovine susceptibility to mastitis, autophagy may reduce mastitis. In addition, autophagy may inhibit inflammation by suppressing the secretion of pro-inflammatory cytokines [90], further indicating that autophagy is associated with inflammatory response. Thus, melatonin may effectively treat mastitis by alleviating oxidative stress and enhancing the progression of autophagy.

## 3. The Effect of Melatonin on Inflammation

Mastitis is a major inflammatory disease, with characteristics common to other inflammatory responses [4]. Inflammatory responses are closely associated with oxidative stress, cytokine release, and mitochondrial dysfunction [87]. Melatonin is reported to act as a potential exogenous pharmacological agent to suppress inflammatory responses, alleviate oxidative stress [91], reduce cytokine release [92], and restore mitochondrial function [93].

Inflammation within the body is generally accompanied by oxidative stress, with increased production of ROS having a severe adverse impact on redox balance [85,87]. Antioxidants can protect cells against elevated oxidative stress conditions. The antioxidative properties of melatonin, a potent free radical scavenger, are caused by both its direct elimination of toxic oxygen derivatives and its ability to enhance the activity of other antioxidants [12]. Melatonin scavenges free radicals by donating an electron or a hydrogen atom, subsequently triggering a series of cascade reactions [94]. The interactions of melatonin with oxygen derivatives produce massive amounts of relevant metabolites, including N^1^-acetyl-N^2^-formyl-5-methoxykynuramine (AFMK) and N^1^-acetyl-5-methoxykynuramine (AMK) [12]. Both AFMK and AMK are powerful free radical scavengers, with more potent capacity to neutralize ROS than melatonin itself [95,96]. Melatonin can also indirectly act as an antioxidant by upregulating the activity of other antioxidant enzymes, such as superoxide dismutase, catalase, and glutathione peroxidase [97].

**Figure 1:**
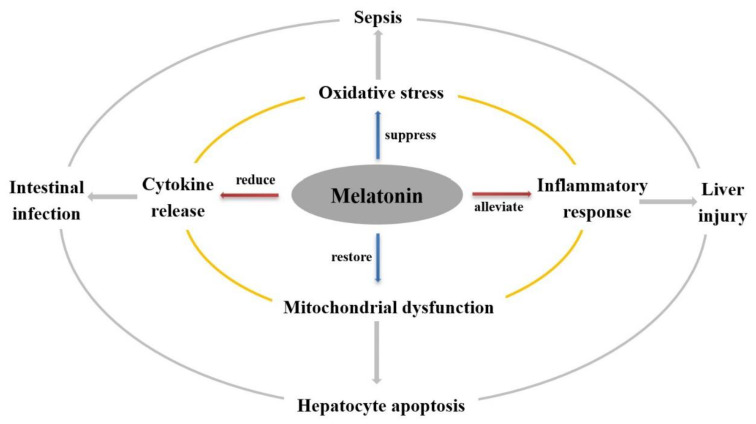
The roles of melatonin in different types of inflammation. Melatonin has already been widely used to treat various types of inflammation. Briefly, melatonin was found to be effective in treating sepsis by suppressing oxidative stress, liver injury by alleviating inflammatory response, hepatocyte apoptosis by restoring mitochondrial dysfunction, and intestinal infection by reducing cytokine release.

Melatonin has already been widely used to treat various types of inflammation. For example, melatonin has protective effects against severe septic shock and septic organ injury induced by bacterial pathogens [98,99]. Melatonin was found to be effective in animal models of sepsis by stimulating various antioxidant enzymes and enhancing antioxidant defenses. Moreover, melatonin has protective effects on mitochondrial function and inhibitory effects on cell apoptosis. For example, melatonin was found to prevent or reduce liver injury by inhibiting oxidation, inflammation, hepatic stem cell proliferation, and hepatocyte apoptosis [100]. Melatonin was also reported to alleviate hepato-intestinal inflammation and alterations in bacterial populations induced by the short-term ingestion of a high-fat diet, as well as reducing ileal inflammation, colonic motility, and perirenal fat accumulation [101]. Moreover, an assessment of the effect of melatonin on intestinal infection using an in vitro model of inflammatory intestinal epithelium found that melatonin modulates the local inflammatory process at the intestinal level, as well as reducing the levels of pro-inflammatory mediators, such as interleukin (IL)-6 and IL-8 [102]. It is well-known that IL-6 and IL-8 are primarily involved in immune and inflammatory responses and play vital roles in the pathophysiology of mastitis [103]. As a result, the promising anti-inflammatory mechanism of melatonin on mastitis could be related to the alleviated effect of melatonin on pro-inflammatory cytokines. In addition, melatonin also inhibits the activation of nuclear factor κ-light-chain-enhancer of activated B cells (NF-κB) and DNA demethylation [104]. When it comes to NF-κB signaling, it plays a critical role in the activation and enhancement of inflammatory responses. Briefly, as the major regulatory transcription factor, it normally exists as homo- or hetero-dimers with p50 and p65 proteins bound to IKB, which is the inhibitor of NF-κB. This complex is sensitive to various factors, such as cytokines, viral and bacterial antigens, free radicals, ultraviolet light, and stress. After being stimulated, IKB kinase activates the phosphorylation of p65 so that inactive NF-κB is activated. Subsequently, activated NF-κB could enter the cell nucleus and further induce the expression of diversely related genes associated with inflammation, adaptive or innate immune response, and cell apoptosis. Combining the key role of the NF-κB signaling pathway in regulating the inflammatory process with the pertinent effect of melatonin on preventing the binding between various factors, as well as NF-κB and its activity to transcribe and translocate into the cell nucleus, it is suggested that the anti-inflammatory function of melatonin is related to the NF-κB signaling pathway [104]. For example, one recent study demonstrated that melatonin could exert an antimicrobial effect and modulate microbial components via NF-κB or other signal transducers and activators of transcription pathways, thereby modulating intestinal immune function along the immune–pineal axis and providing new insights into the use of melatonin in the treatment and management of intestinal diseases (Figure 1) [105]. Hence, similar to other inflammatory conditions, mastitis is accompanied by elevated oxidative stress [102], which suggests that melatonin may be effective in treating mastitis via alleviating or preventing NF-κB signaling pathways.

Melatonin is also involved in the regulation of autophagy [106]. For example, melatonin was shown to stimulate autophagy in matured oocytes of aged mice by upregulating the expression of SIRT1 and microtubule-associated protein 1 light chain 3 (LC3), the concentration of ATP, and decreasing levels of ROS [106]. Sirtuins, a nicotinamide adenine dinucleotide (NAD+)-dependent protein, contain either mono-ADP-ribosyltransferase activity or deacylase activity, including deacetylase, desuccinylase, demalonylase, demyristoylase, and/or depalmitoylase activity [107]. As one of seven isoforms, it was found that SIRT1 occurred both in the cytosol and nucleus of mammals [108]. In addition, SIRT1 broadly participates in various biological processes, such as cell differentiation, autophagy, apoptosis, inflammation, oxidative stress defense, and gene silencing via deacetylating relevant substrate proteins [108,109]. Based on existing studies, several biological effects of melatonin can be exerted through SIRT1-dependent mechanisms involved in the processes of aging, inflammation, and embryo development [106]. It is well-documented that melatonin plays various roles in different cells. For the majority of nontumor cells, it acts as an antioxidant and anti-apoptotic agent to upregulate SIRT1 and plays a pro-oxidant or proapoptotic role under the condition of aging [106]. In other conditions, especially for cancer cells, melatonin inversely exerts an SIRT1-downregulating property [110]. In terms of the existing evidence of melatonin in the role of SIRT1, the specific effects and pertaining molecular mechanisms of melatonin regulating SIRT1 under the condition of mastitis deserve further exploration. LC3, as a typical autophagic protein, is broadly considered to be an autophagic marker and reported to play an important role in the induction and regulation of autophagy as well as the formation of autophagosomes [106]. Therefore, there might be a promising connection between melatonin and autophagy. For example, it is reported that melatonin could attenuate neuronal apoptosis markers or upregulate basal autophagy proteins to exert a protective effect in peripheral sciatic nerves and dorsal root ganglion in oxaliplatin-administered rats [111,112]. Likewise, it was also found that melatonin appeared to promote neuroprotective effects following ischemia/reperfusion-induced brain injury through the effect of activating autophagy in Purkinje cells [113]. Moreover, melatonin promoted the recovery of locomotor function after spinal cord injury by enhancing autophagy and reducing apoptosis [114]. Concerning the above-mentioned references, the ability of melatonin to regulate autophagy, along with the effect of autophagy on mastitis, could provide potential support for the use of melatonin to treat mastitis in future. ATP generation is closely related to the mitochondrion, which is responsible for providing energy within its inner membrane [106]. Therefore, mitochondria are defined as the powerhouse of the cell as they provide the essential energy demanded by a series of physiological processes. Except for the above-mentioned ATP production, they also play a vital role in maintaining intracellular calcium homeostasis, regulating cell apoptosis and formatting ROS [106]. ROS are commonly considered as unstable and highly reactive molecules, which are mainly generated during cellular respiration, especially during the process of mitochondrial oxidative phosphorylation [106,115,116]. Although appropriate numbers of ROS could play an important role in signal transduction, the overproduction of ROS is severely toxic to cells and organs [117]. It was found that excessive ROS production along with the failure of antioxidant defense systems could result in oxidative stress, which subsequently leads to a series of damages to cellular macromolecules, such as nucleic acids, proteins, and lipids, while further inducing the pathogenesis of several diseases, such as mastitis [87]. Therefore, melatonin, known as a potent free radical scavenger and antioxidant, acts as a powerful protector against molecular and tissue injury [86]. Some of the pertained mechanisms by which melatonin operates as an agent to scavenge excessive ROS are associated with melatonin and the mitochondrion. For example, it was found that melatonin could act as a mitochondria-regulating factor involved in the pathophysiology of breast cancer [118]. Some studies revealed that melatonin was related to a reduction in the production of pineal gland-derived circadian melatonin, which is considered a risk factor for breast cancer [119,120]. However, further investigations into the detailed association between melatonin and the mitochondrion of inflammatory mammary cells are needed.

Inflammation is associated with the recruitment of various immune system cells and the activation of immune responses [121,122]. Melatonin was found to affect the immune system by modulating immune responses [123] and interacting with immune system cells [124]. In other words, melatonin participates in mediating immunity, which is partly associated with the crosstalk between the pineal gland and the immune system. As a neuroendocrine organ, the pineal gland can convert environmental photoperiodic information into a biochemical message by virtue of melatonin. Subsequently, melatonin regulates the activity of numerous target tissues after it is released into the bloodstream and arrives in different parts of the body through the circulatory system. Among the numerous actions of melatonin on the immune system, melatonin is an immunomodulator that can regulate the development, differentiation, and function of immune cells via its membrane and nuclear receptors [124]. For instance, melatonin can remarkably regulate neutrophil function based on immune response and the cell migration process. As the first line of defense, neutrophils effectively resist microbial infections by inducing acute inflammatory responses. In addition, the administration of exogenous melatonin was reported to stimulate the migration of immune cells into injured tissues [125], a process that may be associated with the presence of melatonin receptors on the membranes of immune cells [126], especially in lymphocytes. Indeed, T cells express both membrane and nuclear binding sites for melatonin. In addition, T cells also possess the four specific enzymes that are involved in the process of synthesizing melatonin from tryptophan, which indicates that T cells could produce a certain amount of melatonin [127]. Moreover, it was reported that melatonin could regulate the response of helper T cells, including Th1, Th2, Th17, and Tregs, which play an important and intricate role in the immune system. However, the pertaining mechanism is still controversial [127]. For example, melatonin inhibits Th1 responses but stimulates Th2 responses, which implies that melatonin might be involved in regulating the balance of Th1/Th2 cells. Furthermore, the high expression of RORα increases the affinity between Th17 cells and melatonin, which directly affects the activity of Th17 cells [127]. Taken together, melatonin can activate immune reactions under basal, chronic stress, immunosuppressive, and aging conditions, or suppress exacerbated immune responses under conditions of acute inflammation.

## 4. The Effect of Melatonin on Bacteria-Induced Mastitis

**Figure 2:**
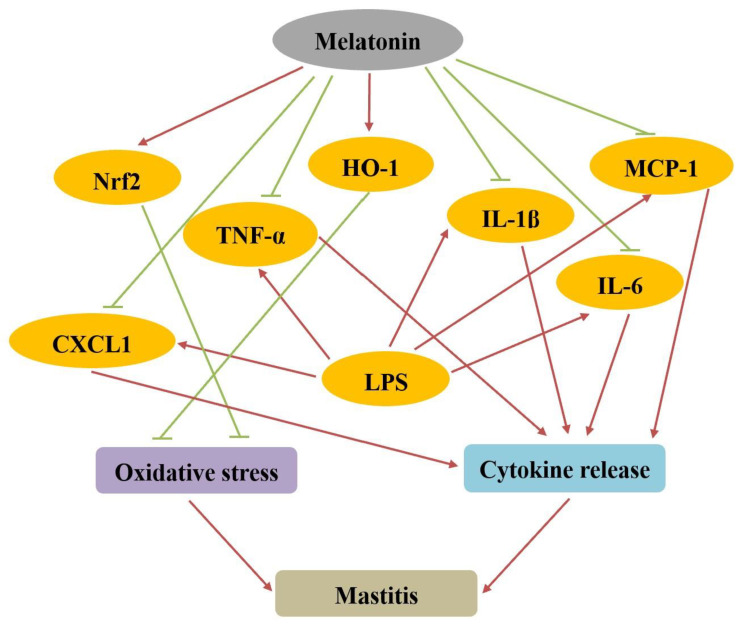
The targets of melatonin to alleviate mastitis based on two recent studies. Melatonin is able to upregulate the expressions of Nrf2 and HO-1, and inhibit the expressions of TNF-α, IL-1β, IL-6, CXCL1, and MCP-1 mRNAs and proteins in LPS-stimulated mastitis. Red arrow represents the stimulatory effect and green line shows the inhibitory effect.

Because melatonin has potent anti-inflammatory effects on different tissues, two recent studies evaluated the effect of melatonin on mammary gland infection (Figure 2). In one study, bovine mammary epithelial cells (bMECs) were treated with lipopolysaccharide (LPS) to induce an inflammatory response [103]. As the component of the outer membrane of Gram-negative bacteria, LPS has been broadly considered to be an endotoxin on account of its capacity to rapidly recruit immune cells and overly elicit several kinds of pro-inflammatory cytokines and chemokines, which impact various organs [128]. Likewise, LPS has also been categorized as a danger-associated molecular pattern and displays a priming function via Toll-like receptor 4 (TLR4) and NF-κB signaling pathways [103]. TLR4 is a pattern recognition receptor which binds to LPS and overexpresses in various inflammatory states triggered by LPS, subsequently activating relevant downstream inflammatory pathways [129]. Melatonin is able to protect bMECs from LPS-induced inflammatory and oxidant stress damage by inhibiting the LPS-binding protein signaling pathway, reducing the expression of pro-inflammatory cytokines induced by LPS, and upregulating the expression of nuclear factor-erythroid 2-related factor 2 (Nrf2) and heme oxygenase-1 (HO-1) in the Nrf2 antioxidant defense pathway [103,130]. Nrf2 is a pleiotropic protein and is generally regarded as a crucial antioxidant sensor [103]. Once it has been activated, Nrf2 passes through the cytoplasm and translocates into the nucleus, further interacting with the antioxidant defense system in facilitating the transcription of target genes, especially HO-1 [130]. Based on the capacity of inducing the transcription, Nrf2 has been identified as a redox-sensitive transcription factor, which could activate a series of transcription of antioxidative, cytoprotective, and anti-inflammatory genes, and successively promote the resistance of oxidative stress and exhibit the protective function against inflammation [103,104]. With respect to melatonin, it could promote the translocation of Nrf2 into the nucleus and stimulate target gene expression [103,131]. Taken together, the promotional effect of melatonin on Nrf2 provides a novel support for applying melatonin as a potential therapeutic candidate to treat oxidative stress and acute inflammation induced by LPS. However, the specific mechanism by which melatonin is associated with an LPS-related pathway has not been determined. Thus, although the actual mechanism of action of melatonin on clinical or subclinical mastitis has not been evaluated, melatonin was able to protect mouse mammary tissue from LPS-induced damage [132,133]. Melatonin inhibited the expression of TNF-α, IL-1β, IL-6, CXCL1, and MCP-1 mRNAs and proteins in LPS-stimulated mouse mammary tissue [132]. Exogenous melatonin administration was found to attenuate bacterial-induced injury. For example, the exogenous administration of melatonin during acute infection with *Staphylococcus aureus* and *Escherichia coli* increased the reduced glutathione content and decreased the enhanced superoxide dismutase activities due to bacterial infection, as well as reducing lipid peroxidation and catalase activities in the liver, brain, and spleen [134]. Melatonin also modulated the overproduction of TNF-α, IL-6, and IFN-γ during acute bacterial infection by reducing neutrophil recruitment to the spleen, as well as modulating iNOS and COX-2 expression in the hypothalamus, suggesting that administration of melatonin could protect against bacterial-induced inflammation. Melatonin was also able to inhibit oxidative stress in bacterial cells, including *Escherichia coli, Staphylococcus aureus*, and *Streptococcus* [135]. Thus, the latent effect of melatonin on bovine mammary gland is worthy of further exploration.

## 5. Conclusions

Because of its vital role in improving antioxidant capacity, resisting bacterial-induced infection, and interacting with the immune response, melatonin can be considered a feasible agent to treat bovine mastitis. Based on the results of many studies that have explored the effects of melatonin on inflammation in different tissues, it will be interesting to determine the importance of melatonin in modulating bovine mammary gland function and immune response. The effects of melatonin on bovine mastitis may be associated with its receptors, both at the membrane and in the nucleus. However, more findings are required for a better understanding of the anti-inflammatory effect of melatonin on different triggered pathways in bacterial-induced mastitis. Several cellular signaling pathways, such as NF-κB and STATs, are associated with the melatonin regulation of inflammatory damage. Various cytoplasmic and mitochondrial binding sites for melatonin may also be involved through receptor-independent signaling pathways. However, the specific cell signaling pathways involved in melatonin regulation of mastitis remain unknown. In addition, few studies to date evaluated the effect of melatonin on mammary gland infection, with most of these performed in mice. Two recent studies showed that melatonin could protect bMECs from LPS-induced inflammation and oxidative stress damage, but to date, no studies have evaluated the effects of melatonin on bovine mastitis. Although additional research is required to determine the effect of melatonin on bovine mammary gland infection, the current findings suggest that melatonin treatment may alleviate bovine mastitis.
